# Exotic Small Mammals as Potential Reservoirs of Zoonotic *Bartonella* spp.

**DOI:** 10.3201/eid1504.081223

**Published:** 2009-04

**Authors:** Kai Inoue, Soichi Maruyama, Hidenori Kabeya, Keiko Hagiya, Yasuhito Izumi, Yumi Une, Yasuhiro Yoshikawa

**Affiliations:** Nihon University, Fujisawa, Kanagawa, Japan (K. Inoue, S. Maruyama, H. Kabeya, K. Hagiya, Y. Izumi); Azabu University, Sagamihara, Kanagawa (Y. Une); The University of Tokyo, Bunkyo-ku, Tokyo, Japan (Y. Yoshikawa)

**Keywords:** Bartonella, emerging infectious diseases, zoonoses, exotic animal, citrate synthase gene, research

## Abstract

To evaluate the risk for emerging human infections caused by zoonotic *Bartonella* spp. from exotic small mammals, we investigated the prevalence of *Bartonella* spp. in 546 small mammals (28 species) that had been imported into Japan as pets from Asia, North America, Europe, and the Middle and Near East. We obtained 407 *Bartonella* isolates and characterized them by molecular phylogenetic analysis of the citrate synthase gene, *gltA*. The animals examined carried 4 zoonotic *Bartonella* spp. that cause human endocarditis and neuroretinitis and 6 novel *Bartonella* spp. at a high prevalence (26.0%, 142/546). We conclude that exotic small mammals potentially serve as reservoirs of several zoonotic *Bartonella* spp.

The genus *Bartonella* includes a variety of gram-negative, fastidious, hemotrophic bacteria that are transmitted by blood-sucking arthropod vectors. The genus consists of 20 species and 3 subspecies; at least 11 of these species are known or suspected to be pathogenic for humans as causative agents of emerging zoonoses (*1*).

The following *Bartonella* spp. have been isolated from wild mice: *B. birtlesii* (*2*), *B. doshiae*, *B. grahamii*, *B. taylorii* (*3*), and *B. vinsonii* subsp. *arupensis* and subsp. *vinsonii* (*4*). In several countries, the following species have been carried by rats of the genus *Rattus*: *B. elizabethae* (*5*), *B. tribocorum* (*6*), *B. phoceensis*, and *B. rattimassiliensis* (*7*). In South Africa, strains genetically related to *B. elizabethae* also have been isolated from mice of the genera *Aethomys* and *Tatera* (*8*). The main reservoir of *B. washoensis* is considered to be wild squirrels (*9*). Of these rodent-associated *Bartonella* spp., *B. elizabethae*, *B. grahamii*, *B. vinsonii* subsp. *arupensis*, and *B. washoensis* have been implicated in the human infections endocarditis (*10*), neuroretinitis (*11*), pyrexia and endocarditis (*4*,*12*), and myocarditis (*9*), respectively.

Previous studies have demonstrated high prevalence of infection with *Bartonella* spp. in wild and peridomestic small animals in Europe (*7*,*13*–*15*), North and South America (*5*,*16*–*19*), Asia (*20*–*23*), and Africa (*8*). Thus, these animals are thought to be reservoirs of several *Bartonella* spp. and sources of infection for humans.

Many exotic animals are traded as pets around the world and have been imported into Japan without quarantine. However, no data exist on the prevalence of infection with *Bartonella* spp., especially in exotic pet animals. Our study objectives were to 1) examine the prevalence of *Bartonella* spp. infection in exotic small mammals imported into Japan from various countries, 2) compare the diversity of these *Bartonella* strains by analyzing the partial sequence of the citrate synthase gene (*gltA*), and 3) evaluate the possibility that these mammals may serve as potential reservoirs of zoonotic *Bartonella* spp.

## Materials and Methods

### Animals and Samples

For this study, 546 exotic small mammals were purchased from trading companies. The animals represented 3 orders and included 6 families, 23 genera, and 28 species (Table 1). They had been imported into Japan as pets from June 2004 through October 2007 from 8 countries in 4 geographic regions: Asia (China, Thailand, and Indonesia), Europe (the Netherlands and Czech Republic), North America (United States), and the Middle and Near East (Egypt and Pakistan). Of the 546 animals, 367 had been captured in their natural environment and 179 had been bred in the exporting countries. Heparinized blood samples were aseptically collected from each animal (anesthetized with chloroform) and centrifuged at 3,000 rpm for 15 min. Plasma was removed and the blood sample pellets were kept at –80ºC until examination.

**Table 1:** Prevalence of *Bartonella* spp. among exotic small mammals imported into Japan as pets, June 2004–October 2007

Origin	Animal, taxonomic species	No. positive/no. tested (%)	Subtotal (%)
Wild-captive			
Asia			
China	Spermophilus dauricus*	4/10 (40.0)	42/89 (47.2)
	*Sciurus vulgaris* subsp. orientis*	2/10 (20.0)	
	Tamias sibiricus*	12/29 (41.4)	
	Pteromys volans*	5/10 (50.0)	
Thailand	Callosciurus notatus*	19/30 (63.3)	
North America			
USA	Tamiasciurus hudosonicus*	3/18 (16.7)	27/68 (39.7)
	Glaucomys volans*	6/10 (60.0)	
Unknown	Sp. columbianus*	6/20 (30.0)	
	Sp. richardsonii*	12/20 (60.0)	
Europe			
The Netherlands	*Pachyuromys duprasi†*	13/18 (72.2)	13/47 (27.7)
The Netherlands, Czech Republic	*Octodon degus*‡§	0/29 (0.0)	
Middle and Near East			
Egypt	*Mus minutoides†*	0/20 (0.0)	55/163 (33.7)
	*Acomys cahirinus†*	3/31 (9.7)	
	*A. russatus†*	8/13 (61.5)	
	*Lemniscomys barbarus†*	0/11 (0.0)	
	*Psammomys obesus†*	6/10 (60.0)	
	*Meriones tristrami†*	0/4 (0.0)	
	*Sekeetamys calurus†*	10/10 (100)	
	*Gerbillus pyramidum†*	9/10 (90.0)	
	*Jaculus orientalis*¶	13/16 (81.3)	
	*J. jaculus*¶	6/8 (75.0)	
	*Hemiechinus auritus#*	0/10 (0.0)	
Pakistan	*Salpingotulus michaelis*¶	0/20 (0.0)	
	Subtotal	137/367 (37.3)	
Breeder facility			
Asia			
China	Tamias sibiricus*	5/30 (16.7)	5/60 (8.3)
Indonesia	Petaurus breviceps**	0/20 (0.0)	
Thailand	Pe. breviceps**	0/10 (0.0)	
Europe			
The Netherlands	*Lagurus lagurus†*	0/9 (0.0)	0/99 (0.0)
	*Pa. duprasi†*	0/10 (0.0)	
	*Mesocricetus auratus†*	0/20 (0.0)	
	*Phodopus roborovskii†*	0/10 (0.0)	
The Netherlands, Czech Republic	*Ph. sungorus*†‡	0/30 (0.0)	
	*O. degus*‡§	0/20 (0.0)	
Middle and Near East			
Pakistan	*Sa. michaelis*¶	0/20 (0.0)	0/20 (0.0)
	Subtotal	5/179 (2.8)	
	Total	142/546 (26.0)	

*Member of the order Rodentia, family Sciuridae. †Member of the order Rodentia, family Muridae. ‡Data for the Netherlands and Czech Republic are pooled because number of animals from these 2 countries was unknown. §Member of the order Rodentia, family Octododidae. ¶Member of the order Rodentia, family Dipodidae. #Member of the order Insectivora, family Erinaceidae. **Member of the order Diprotodonia, family Petauridae.

### Isolation of Bacteria

The blood sample pellets were thawed at room temperature, 100-μL supplemented Medium 199 (*24*) was added to each pellet, and each sample was mixed well. A 100-μL sample of each mixture was plated on 2 heart infusion agar (DIFCO, Sparks Glencoe, MI, USA) plates containing 5% defibrinated rabbit blood. The plates were incubated at 35ºC under 5% CO_2_. After 2 weeks of incubation, 2 or 3 colonies with genus *Bartonella* morphologic characteristics (small, gray or cream-yellow, round colonies) were picked from each plate, confirmed to be gram-negative pleomorphic bacteria, and subcultured using the same conditions used for the original cultures.

### DNA Extraction and PCR

The genomic DNA of each isolate was extracted by using InstaGene Matrix (Bio-Rad, Hercules, CA, USA). The extracted DNA was used for PCR analysis of a 312-bp part of the *gltA* gene to confirm that the bacteria were from the genus *Bartonella*. PCR was performed by using an iCycler (Bio-Rad) with a 20-μL mixture containing 20 ng extracted DNA, 200 μM of each deoxynucleoside triphosphate, 1.5 mmol/L MgCl_2_, 0.5 U Taq DNA polymerase (Promega, Madison, WI, USA), and 1 pmol of each primer. The specific primer pair and PCR conditions for *gltA* were as previously reported (*25*).

### DNA Sequencing and Phylogenetic Analysis

The PCR products were purified by using a commercial kit (Spin Column PCR Products Purification Kit; Bio Basic, Markham, Ontario, Canada). Direct DNA sequencing of the purified PCR products was carried out by using dye terminator chemistry with specific primers (*25*) and a Model 3130 Genetic Analyzer (Applied Biosystems, Foster City, CA, USA). The 312-bp *gltA* sequences from the isolates and type strains of established *Bartonella* spp. in GenBank/EMBL/DDBJ were aligned with the Clustal X program (*26*), and a phylogenetic tree was drawn, based on the sequence data and using the neighbor-joining method (*27*) with the Kimura 2-parameter distance method (*28*) in MEGA 4 (*29*). Bootstrap analysis was carried out with 1,000 replications (*30*).

### Statistical Analysis

The results were analyzed in 2×2 tables. Chi-square tests were used to examine the statistical significance; p<0.05 was considered significant.

## Results

### Prevalence of Bartonellae

The prevalence of bartonellae in the exotic small mammals examined was 26.0% (142/546). A total of 407 isolates were obtained from the 142 bacteremic animals (Table 1). The prevalence by animal origin was 37.3% (137/367) in captive animals and 2.8% (5/179) in animals from breeder facilities. A significantly higher prevalence of bartonellae was observed in captive animals than in animals from breeder facilities (p<0.001). In the captive animals, the prevalence by region varied up to 47.2% in Asia, which is higher than the 39.7% prevalence in North America. The prevalence of bartonellae by corresponding taxonomic family of host animal ranged from 38.6% (49/127) in the family Muridae to 43.9% (69/157) in the family Sciuridae. No bartonellae were detected in animals in the families Octodontidae and Erinaceidae. Among animals from breeders, only 5 chipmunks (*Tamias sibiricus*) from China were found to be infected with bartonellae; no bartonellae were isolated from animals in the families Petauridae, Muridae, Octodontidae, or Dipodidae.

Bartonellae were isolated from 17 of the 28 animal species studied (Table 1). The prevalence by animal species varied from 9.7% (3/31) in the Cairo spiny mouse (*Acomys cahirinus*) to 100% (10/10) in the bushy-tailed jird (*Sekeetamys calurus*). Prevelances were considerably higher for the bushy-tailed jird, large Egyptian gerbil (*Gerbillus pyramidum*), greater Egyptian jerboa (*Jaculus orientalis*), and lesser Egyptian jerboa (*J. jaculus*) at 100% (10/10), 90.0% (9/10), 81.3% (13/16), and 75.0% (6/8), respectively.

### DNA Sequences and Phylogeny of Isolates

The 407 isolates in this study were classified into 53 genotypes on the basis of DNA sequence analysis of a 312-bp fragment of their *gltA* genes. The sequence of a genotype from a Cairo spiny mouse was identical to that of the *B. elizabethae* type strain (GenBank accession no. Z70009) isolated from a human patient with endocarditis (*10*). The other 52 genotypes were found to be novel genotypes after comparison with known *Bartonella* spp. The phylogenetic tree of the *gltA* sequences shows that the 52 novel genotypes are clearly clustered in 10 genogroups, designated A to J (Figure).

**Figure:**
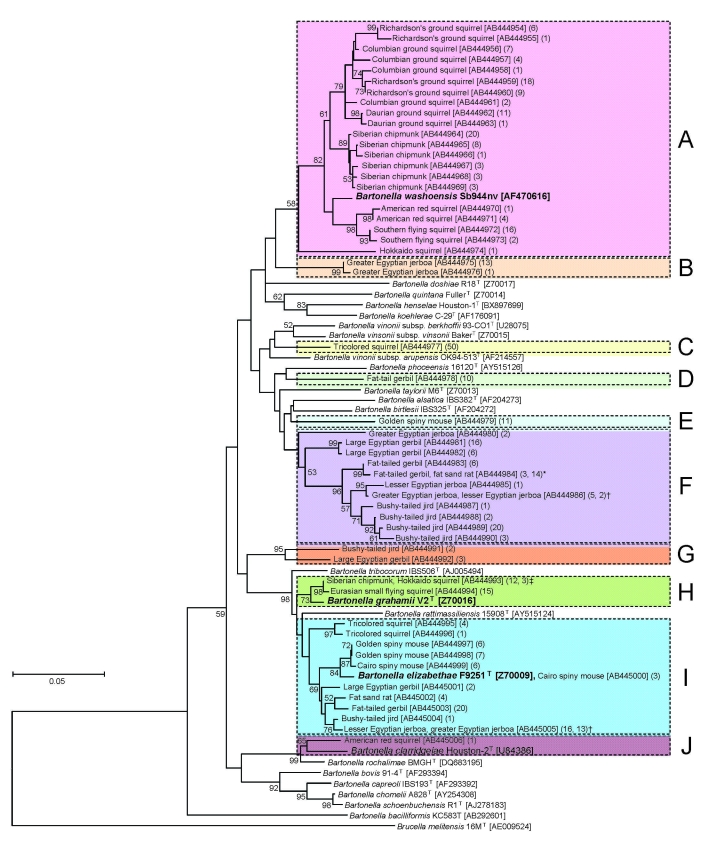
Phylogenetic tree based on a 312-bp region of the citrate synthase (*gltA*) gene sequence, constructed from *Bartonella* spp. isolates from 142 exotic small mammals imported into Japan as pets, June 2004–October 2007.Isolates from imported animals were compared with the type strains of known *Bartonella* spp. The phylogenetic tree was constructed by the neighbor-joining method, and bootstrap values were obtained with 1,000 replicates if values >50% were noted. The *Brucella melitensis* strain 16M sequence was used as an out-group. The GenBank accession number and the number of isolates are indicated in brackets and parentheses, respectively. The scale bar indicates 0.05 estimated nucleotide substitutions per site. Each colored column corresponds to genogroup A to J. Isolates showing identical genotypes were obtained from fat-tailed gerbils and fat sand rats (*), greater Egyptian jerboas and lesser Egyptian jerboas (†), and Siberian chipmunks and Hokkaido squirrels (‡).

Of the 52 novel genotypes, genogroup A, which consisted of 21 genotypes (AB444954 to AB444974) isolated from 7 squirrel species, was related to *B. washoensis* strain Sb944nv (AF470616), which was isolated from a California ground squirrel (*Spermophilus* *beecheyi*) and was genetically identical to an isolate from a human patient with myocarditis (*9*). The sequence similarities of these genotypes and *B. washoensis* strain Sb944nv ranged from 94.2% to 97.4%. Genogroup A contained *B. washoensis*–like genotypes; the genotypes from each squirrel species formed a separate clade, except for the genotypes from Richardson’s ground squirrels (*Sp. richardsonii*) and Columbian ground squirrels (*Sp. columbianus*), which formed a mixed clade (Figure).

In this study, 18 genotypes formed the 6 unique genogroups B to G. The DNA sequences of the genotypes in each genogroup showed relatively low similarity (82.4%–94.6%) to the type strains of known *Bartonella* spp., and sequence similarities between genogroups B to G were also low (87.5%–93.6%). The novel *Bartonella* genogroups B, C, D, and E were isolated from greater Egyptian jerboas, tricolored squirrels (*Callosciurus notatus*), fat-tailed gerbils (*Pachyuromys duprasi*), and golden spiny mice (*A. russatus*), respectively. The genotypes in group F were isolated from 6 animal species: large Egyptian gerbils, fat-tailed gerbils, fat sand rats (*Psammomys obesus*), lesser Egyptian jerboas, greater Egyptian jerboas, and bushy-tailed jirds; those in genogroup G were isolated from a bushy-tailed jird and a large Egyptian gerbil (Figure). In genogroup F, 3 of the 9 isolates from fat-tailed gerbils and the 14 isolates from fat sand rats had identical *gltA* DNA sequences. Furthermore, 5 of the 7 isolates from greater Egyptian jerboas and 2 of the 3 isolates from a lesser Egyptian jerboa also had identical sequences.

The 2 novel genotypes (AB444993 and AB444994) in genogroup H were also isolated from Siberian chipmunks, a Hokkaido squirrel (*Sciurus vulgaris* subsp. *orientis*), and Eurasian small flying squirrels (*Pteromys volans*). Their sequences showed high similarity (98.4%–98.7%) to *B. grahami* type strain (V2) (Figure).

The 10 novel genotypes in genogroup I were isolated from 9 animal species, and the sequence similarities between the genotypes (AB444995 to AB445005) and *B. elizabethae* type strain (F9251) ranged from 95.5% to 98.7%. The DNA sequences of *gltA* of the 3 isolates from a Cairo spiny mouse (AB445000) were identical to that of *B. elizabethae* (F9251). The sequences of the 13 isolates from lesser Egyptian jerboas were identical to those of the 16 isolates from greater Egyptian jerboas.

In genogroup J, a unique genotype (AB445006) was isolated from an American red squirrel (*Tamiasciurus hudosonicus*); it had 96.2% sequence similarity to *B. clarridgeiae* type strain (Houston-2), whose natural reservoir is cats (Figure).

### Multiple Infections with Different *Bartonella* Genogroups and Genotypes

Of the 142 *Bartonella*-positive animals, 25 (17.6%) were found to be infected with different *Bartonella* genogroups or genotypes (Table 2). A lesser Egyptian jerboa carried 3 different genotypes in 2 genogroups; the other 24 animals carried 2 different genogroups or genotypes. Of these 24 animals, an American red squirrel carried a *B. washoensis*–like strain in genogroup A and *B. clarridgeiae*–like strains in genogroup J; 11 animals were infected with *B. elizabethae*–like strains in genogroup I and strains in genogroups B, C, D, E, F, or G, and the remaining 12 carried different genotypes in the same genogroup (Table 2).

**Table 2:** Multiple infection of different *Bartonella* genotypes in exotic small mammals imported into Japan as pets, June 2004–October 2007

Host	No. animals	GenBank accession nos. of the isolates in 9 genogroups*								
		A	B	C	D	E	F	G	I	J
Daurian ground squirrel	1	4962 4963								
Siberian chipmunk	1	4965 4966								
	1	4964 4965								
Tricolored squirrel	2			4977					4995	
	1			4977					4996	
American red squirrel	1	4971								5006
Southern flying squirrel	1	4972 4973								
Columbian ground squirrel	1	4957 4958								
Richardson's ground squirrel	2	4959 4960								
	1	4954 4959								
	1	4954 4955								
Fat-tailed gerbil	3				4978				5003	
Golden spiny mouse	1					4979			4998	
Fat sand rat	1						4984		5002	
Bushy-tailed jird	1						4988 4989			
	1							4991	5004	
	1						4987 4989			
Large Egyptian gerbil	1						4981		5001	
Greater Egyptian jerboa	1		4975 4976							
	1						4986		5005	
Lesser Egyptian jerboa	1						4986 4985		5005	

*GenBank accession numbers all begin with AB44 and are abbreviated to the last 4 digits; e.g., AB444962 appears as 4962.

## Discussion

We report prevalence of bartonellae in exotic small mammals imported into Japan as pets. We found that 26.0% (142/546) of the animals examined had bartonellae in their blood. Prevalence among wild captive animal species was high (37.3%), significantly higher (p<0.001) than that among animals from breeder facilities. Of the 179 animals (representing 9 species) from breeder facilities, only 5 Siberian chipmunks imported from a Chinese breeder were found to carry bartonellae, and these were of the same genotype as bartonellae from wild captive animals. These results suggest that animals in breeder facilities may be maintained under hygienic conditions from birth to export, so they rarely have contact with wild animals or blood-sucking arthropod vectors.

Most isolates from animals in the family Sciuridae (58.7%; 122/208) were in genogroup A and showed high sequence similarity to *B. washoensis*. Kosoy et al. (*9*) have reported that *B. washoensis* is widely distributed in ground squirrels in the western part of the United States and that it was isolated from a human with myocarditis in Nevada, USA. Thus, captive squirrels carrying *B. washoensis*–like organisms could serve as a source of infection for humans.

Animals in the family Sciuridae were also found to be carrying several genotypes of bartonellae in genogroups C, *B. grahamii*–like strains in genogroup H, *B. elizabethae*–like strains in genogroup I, and *B. clarridgeiae*–like strains in genogroup J. The sequence similarities between the genotypes and the related *Bartonella* spp. type strains ranged from 98.4% to 98.7% for *B. grahamii*, from 95.5% to 95.8% for *B. elizabethae*, and were 96.2% for *B. clarridgeiae*. In humans, *B. grahamii*, *B. elizabethae*, and *B. clarridgeiae* have been reported to cause neuroretinitis (*11*), endocarditis (*10*), and cat-scratch disease (*31*), respectively. These findings suggest that exotic squirrels also might be a potential source of *Bartonella* infections in humans. Although *B. clarridgeiae* has mainly been isolated from cats (*1*), *B. clarridgeiae*–like strains were isolated from an American red squirrel in this study. *B. clarridgeiae*–like organisms have also been isolated from yellow-necked mice (*Apodemus* *flavicollis*) in Sweden (*14*) and Greece (*15*).

The sequence similarity of the *gltA* sequence (312 bp) of the *B. clarridgeiae*–like genotype isolated in our study to that of the strain isolated from the yellow-necked mouse (AF391788) was relatively high (97.7%). Recently, *B. rochalimae*, a *B. clarridgeiae*–like organism, was isolated from a human patient with bacteremia, fever, and splenomegaly (*32*). The *B. clarridgeiae*–like strain from the American red squirrel in this study also showed high *gltA* sequence similarity (96.8%) with that of *B. rochalimae* strain BMGH. Studies will be required to clarify the pathogenicity of *B. clarridgeiae*–like organisms for humans. Such studies would include 1) evaluation of the organisms’ ability to invade human erythrocytes and/or endothelial cells, 2) demonstration of the presence and expression of the genes of type 4 secretion systems (VirB/VirD4 or Vbh) and Trw, and 3) comparisons of the entire genome sequences of the organisms and with those of other human pathogenic *Bartonella* spp.

In this study, *Bartonella* genogroups D, E, and G were isolated from animals in the family Muridae, and *Bartonella* genogroup B was isolated from animals in the family Dipodidae. These findings suggest strict host specificity between the strains in these genogroups and the host animal family. However, findings also showed wide host species diversity; strains in genogroup F were isolated from 6 animal species, and strains from genogroup I (*B. elizabethae*–like) were isolated from 9 animal species. *Bartonella* strains in genogroup F were isolated from animals in the families Muridae and Dipodidae. Genogroup I (*B. elizabethae*–like) strains were also isolated from animals in the family Sciuridae. *B. elizabethae* has been isolated from different animal species, e.g., a human patient and genus *Rattus* rats (*5*,*10*), and *B. elizabethae* DNA has been isolated from a dog (*33*). In our study, 3 *Bartonella* isolates from a Cairo spiny mouse imported from Egypt had an identical *gltA* sequence to that of the *B. elizabethae* type strain. Thus, some *Bartonella* spp., such as *B. elizabethae* and *B. washoensis*, infect host animals in diverse families and may have zoonotic potential.

In the present study, 17.6% (25/142) of exotic animals were infected with different *Bartonella* genotypes or genogroups. In particular, 3 isolates from a greater Egyptian jerboa were classified in 3 different genotypes. Of the 25 *Bartonella*-positive animals, 13 showed co-infection with different *Bartonella* genogroups. Of these 13 animals, 12 carried *B. elizabethae*–like strains in genogroup I. In contrast, strains with identical *gltA* sequences were isolated from 2 different animal species, such as greater Egyptian jerboas and lesser Egyptian jerboas, Siberian chipmunks and Hokkaido squirrels, and fat-tailed gerbils and fat sand rats. These findings suggest that some *Bartonella* species have a wide host range and may be transmitted horizontally by some blood-sucking arthropod vectors with low host specificity.

In summary, we examined the possibility that exotic small mammals may be reservoirs of zoonotic *Bartonella* spp. around the world. The animals in this study carried, at high prevalence, several *Bartonella* spp. that are human pathogens. Novel species were suggested by the fact that some of the genotypes in 6 genogroups (B to G) showed relatively low similarity (<94.6%) to known *Bartonella* spp. and formed independent clusters according to phylogenetic analysis based on partial *gltA* sequences. More taxonomic studies should sequence other housekeeping genes, such as *rpoB*, 16S rRNA, *ftsZ*, *groEL*, and *ribC,* to confirm whether these isolates are novel *Bartonella* spp. (*34*). To prevent human infections by *Bartonella* spp. carried by exotic small mammals, a quarantine system for these animals should be established as early as possible. Further studies will be necessary to clarify the route of transmission among exotic small mammals and to evaluate the pathogenicity for humans and animals of the isolates belonging to novel *Bartonella* genotypes found in this study.
